# Aberrant Resting-State Functional Connectivity Density in Patients with Hepatitis B Virus-Related Cirrhosis

**DOI:** 10.1155/2016/4168512

**Published:** 2016-06-14

**Authors:** Xiao-Fei Lv, Hua-Wang Wu, Li Tian, Lu-Jun Han, Jing Li, Ying-Wei Qiu, Gui-Hua Jiang, Xue-Lin Zhang, Rong Zhang, Chuan-Miao Xie

**Affiliations:** ^1^Department of Medical Imaging, Sun Yat-sen University Cancer Center, State Key Laboratory of Oncology in South China, Collaborative Innovation Center for Cancer Medicine, Guangzhou 510060, China; ^2^The Affiliated Brain Hospital of Guangzhou Medical University (Guangzhou Huiai Hospital), Guangzhou 510370, China; ^3^Department of Medical Imaging, Guangdong No. 2 Provincial People's Hospital, Guangzhou 510317, China; ^4^Medical Imaging Center, Nanfang Hospital, Southern Medical University, Guangzhou 510515, China

## Abstract

There is increasing evidence that cirrhosis may affect functional connectivity among various brain regions in patients prior to onset of overt hepatic encephalopathy (HE). However, most investigators have focused mainly on alterations in functional connectivity strengths, and the changes in functional connectivity density (FCD) are largely unknown. Here, we investigated alterations in resting-state FCD in patients with hepatitis B virus-related cirrhosis (HBV-RC) without overt HE. Totally, 31 patients with HBV-RC without overt HE and 30 age- and sex-matched healthy controls underwent resting-state functional MRI examinations. FCD mapping was employed to compute local and global FCD maps. Then, short-range and long-range FCD values were calculated and voxel-based comparisons were performed between the two groups. The HBV-RC group showed significant decreases in FCD, including decreased short-range FCDs in the bilateral middle cingulum gyrus/precuneus, the bilateral cuneus, and the left lingual gyrus/inferior occipital gyrus and decreased long-range FCD in the bilateral cuneus/precuneus. In addition, the decreased long-range FCD in the bilateral cuneus/precuneus in the HBV-RC group was related to performance on the psychometric hepatic encephalopathy score (PHES) test. These findings suggest aberrant functional connectivity density in cirrhotic patients prior to overt HE onset, which may provide better insight into understanding the pathophysiological mechanisms underlying the cirrhotic-related cognitive impairment.

## 1. Introduction

Hepatitis B virus infection remains a serious public health problem, with more than 350 million people chronically infected worldwide [1]. Annually, about 1.0–2.4% of these patients proceed to hepatitis B virus-related cirrhosis (HBV-RC) [1]. Hepatic encephalopathy (HE) is the most serious neuropsychiatric complication of end-stage HBV-RC [1, 2]. They always suffer from a broad spectrum of mental disorders ranging from subtle cognitive dysfunction to stupor and coma [2]. Clinically, cirrhotic patients with overt HE usually exhibit a spectrum of recognizable clinical syndromes on neuropsychiatric examination, while cirrhotic patients without overt HE always present as essentially normal [3]. However, increasing evidence indicates that various degrees of neurocognitive deficits are common, even in patients without any signs of overt HE [3, 4]. These neurocognitive dysfunctions are increasingly relevant because they are associated with poorer quality of life [5], deterioration of daily function [6], and poor prognosis [7]. However, the pathophysiological mechanisms underlying these deficits are still not fully understood.

Resting-state functional MRI (rs-fMRI) has recently attracted considerable attention for investigating brain function in clinical populations. A number of rs-fMRI studies [8–11] have revealed that the pathophysiology of cirrhotic-related cognitive impairment may be associated with alterations in spontaneous low-frequency (<0.08 Hz) blood oxygen level dependent (BOLD) fluctuations. Previous rs-fMRI studies [8–11] with various algorithms, such as regional homogeneity and amplitude of low-frequency fluctuation, have been applied to investigate cirrhosis-related changes in localized brain regions. Those findings [8–11] indicate diffuse abnormalities in intrinsic brain activity in cirrhotic patients. This important information provided by such univariate methods has revealed the roles played by each brain region in the pathogenesis of neurocognitive dysfunction in cirrhotic patients but does not enable evaluations of the interactions among different brain regions.

Resting-state functional connectivity strength (rsFCS) has been developed to measure the temporal correlations of spontaneous fluctuations in brain activity between spatially remote regions. It has been extensively applied to explore the functional interactions among brain regions. rsFCS is increasingly used to investigate the abnormalities of functional integrations in cirrhotic patients among a series of brain areas [12, 13], specific subnetworks (such as the default mode network (DMN), the dorsal attention network, the visual network, and the auditory network) [14, 15], and even the whole-brain network [16]. These studies [12–16] have suggested aberrant functional connectivity in some specific brain regions and networks. They have contributed to the elucidation of the pathophysiological mechanism underlying cognitive deficits in cirrhotic patients. However, nearly most of the previous studies have been concerned with rsFCS changes in cirrhotic brains; the changes in the number of functional connections of each voxel across the whole brain remain unclear for cirrhotic patients.

Resting-state functional connectivity density (FCD) mapping has recently been developed to measure the functional connection number of a given voxel with all other voxels within the whole brain [17–19]. FCD is an ultrafast and unbiased approach that does not rely on a priori selection of the seed regions. It enables the identification of functional connectivity hubs (regions that are connected densely) highly effectively [17–19]. Furthermore, FCD can discriminate among short- and long-range FCD hubs according to the neighboring relationship between different brain voxels [17–19]. The greater the number of brain voxels functionally connected to other voxels, the higher the FCDs they have, and the voxels with higher FCDs are likely to play more important roles in brain information processing [17]. FCD has been successfully used to identify connectivity alterations in aging [20] and some neuropsychiatric disorders [21–23], and it may provide better insights into the pathophysiology of cognitive alterations in cirrhotic patients.

Therefore, the present study used FCD mapping analysis to investigate abnormal connectivity in patients with HBV-RC without overt HE. We aimed to find regions exhibiting altered FCD in patients with HBV-RC. Furthermore, we also investigate whether the cognitive abnormalities in patients with HBV-RC without overt HE were related to these changes in FCD.

## 2. Materials and Methods

### 2.1. Participants

This prospective study was approved by the local Medical Research Ethics Committee. Written informed consent was obtained from each participant prior to the study. Totally, sixty-one right-handed patients, including 31 patients with chronic HBV-related liver cirrhosis without overt HE (26 male and 5 women; mean age: 44.61 ± 10.01 years) hospitalized at our hospital and 30 normal controls (25 male and 5 women; mean age: 43.73 ± 9.32 years) matched for age, sex, and education, were enrolled in this prospective study. Each subject has been clinically proven HBV-RC and the final diagnosis was determined by biopsy or based on clinical examination or biochemical and medical imaging findings. The liver functional status of each patient was assessed according to the Child-Pugh score. The clinical characteristics of all subjects are summarized in Table 1.

Patients were excluded if they presented any current or previous symptoms of overt HE, diffuse hepatocellular carcinoma, or other serious complications (such as gastrointestinal hemorrhage or bacterial infection). The exclusion criteria for all participants also included age younger than 18 or older than 70 years, psychiatric or neurological diseases, severe organic diseases (such as progressive cardiovascular, pulmonary, or renal disorders), severe endocrine or metabolic diseases (such as diabetes and hyperthyroidism), history of drug and alcoholism abuse, history of severe head trauma, infection with other viral hepatitis or human immunodeficiency virus, or any obvious focal lesion detected on conventional brain MRI examination.

### 2.2. Neuropsychological Tests

The PHES encompassing the number connection tests A and B (NCT-A and NCT-B), the digit symbol test (DST), the serial dotting test (SDT), and the line tracing test (LTT) has been recommended by guidelines to evaluate the degree of neurocognitive impairment in cirrhotic patients. Because some of the participants were not so familiar with English alphabets, a Chinese version of the PHES battery reported in a previous study [8] was applied. All participants completed the PHES test battery after an appropriate demonstration and explanation. The final PHES value was calculated as the sum of the scores on the five tests. It ranged between +5 and −15. The method about how to calculate PHES values has been documented in detail elsewhere [8].

### 2.3. MRI Data Acquisition

All MR imaging data were acquired with a 1.5T MR scanner (Achieva Nova Dual; Philips Medical Systems, Best, Netherlands), using a 16-channel neurovascular (NV) coil. Foam padding was used to limit head movement. Before the rs-fMRI scan, conventional MR imaging sequences including T_1_-weighted images [repetition time (TR) = 600 ms, echo time (TE) = 29 ms] and T_2_-FLAIR images [TR = 6000 ms, TE = 120 ms, and inversion time (TI) = 2000] were performed in every participant to detect clinically silent lesions. During rs-fMRI scan, participants were instructed simply to rest with their eyes closed, to remain as motionless as possible, to not think of anything in particular, and to not fall asleep. Whole-brain rs-fMRI data were acquired by using an echo planar imaging (EPI) sequence with the following parameters: TR = 3000 ms, TE = 50 ms, flip angle = 90°, field of view (FOV) = 23 × 23 cm^2^, matrix = 64 × 64, and 4.5 mm slice thickness with no gap. Each brain volume comprised 33 axial slices, and 160 image volumes were collected in each functional run. Each rs-fMRI scan lasted 8 minutes. After the MR examination, each participant was asked some questions to check the degree of collaboration.

### 2.4. Data Preprocessing

The rs-fMRI data were preprocessed with Statistical Parametric Mapping software (SPM8, http://www.fil.ion.ucl.ac.uk/spm/). The first ten volumes of each participant were discarded to ensure magnetization equilibrium and allow the participants to adapt to the environment. The remaining 150 volumes of images were slice-timing-corrected for the acquisition time delays between slices and then realigned to correct the motion between time points. Any participant whose head motion exceeded 1.5 mm in transition or/and 1.5 degrees in rotation was excluded in the following steps [24]. Recent studies have demonstrated that severe head motion can significantly influence the estimation of functional connectivity [25, 26]; therefore, frame-wise displacement (FD) values, which indexes the volume-to-volume changes in head position, were also calculated [25]. The average FD of all subjects was less than 0.3, and there were no significant differences in mean displacement between HBV-RC patients and healthy controls (FD: 0.127 ± 0.061 for HBV-RC patients and 0.143 ± 0.074 for healthy controls; *p* = 0.377) using a two-sample *t*-test (two-tailed). All realigned rs-fMRI images were then spatially normalized into the Montreal Neurological Institute (MNI) template using the standard EPI template and resliced with voxel size of 3 × 3 × 3 mm^3^. Then, six estimated motion parameters and linear drift were removed from all preprocessed data by linear regression. Finally, a temporal band-pass filter (0.01–0.10 Hz) was conducted to reduce very low-frequency drifts and physiological high-frequency noise.

### 2.5. FCD Mapping Calculation

The FCD calculations of each voxel were conducted by using an in-house script written on a Windows 7 platform and based on the method described by Tomasi and Volkow [17–19]. The calculations of FCD were restricted to voxels within the whole-brain gray matter mask [19]. Pearson's linear correlation was applied to evaluate the strength of the functional connectivity between two voxels. If their Pearson correlation coefficient (*R*) is >0.6, the two voxels were considered significantly functionally connected. The global FCD of a given voxel (*x*_0_) was defined as the number of significant functional connections between the voxel and all other voxels across the whole brain [18]. For the short-range FCD of *x*_0_, it was defined as the number of elements between *x*_0_ and its neighbor voxels. For the long-range FCD of *x*_0_, it was equated with the global FCD minus the short-range FCD to isolate the number of nonneighboring functional connections. To reduce the effect of variability across subjects, the short- and long-range FCD maps of each individual were then normalized to their average strength across the whole brain, respectively. Finally, the normalized FCD maps were spatially smoothed with a Gaussian kernel of 8 × 8 × 8 mm^3^.

### 2.6. Statistical Analyses

Two-sample *t*-tests were applied to compare the group differences in age, years of education, and number of cigarettes smoked daily between cirrhotic patients and healthy subjects. The group differences in PHES between the two groups were compared using Mann-Whitney *U* tests. All analyses were performed using SPSS (version 16.0, SPSS Inc., Chicago, IL, USA). A *p* value < 0.05 was deemed statistically significant.

The voxel-wise comparisons of short and long-range FCDs between cirrhotic patients and healthy subjects were conducted by using a one-way analysis of covariance (ANCOVA) with the gender, age, and average FDs as nuisance covariates. Correction for multiple comparisons was performed using the topological false discovery rate (FDR) criterion [27], resulting in a corrected threshold of *p* < 0.05.

The FCDs in clusters with significant differences were extracted and analyzed using region of interest (ROI). Each ROI was a 9 mm isotropic cube including 27 voxels (0.73 cm^3^) centered at the MNI coordinates of the local maxima. To determine if short- and long-range FCDs varied with the progression of cognitive changes in HBV-RC patients, Spearman correlation analyses were used to assess the performance level on the PHES test and the short- and long-range FCD of the ROIs obtained from clusters. Significant group differences were assessed using SPSS.

## 3. Results

### 3.1. Demographic and Cognitive Testing

We found there were no significant differences in age, years of education, and number of cigarettes smoked daily between patients with HBV-RC and healthy controls. However, compared with the healthy controls, the patients with HBV-RC performed significantly poorly on PHES battery of tests (Table 1).

### 3.2. Between-Group FCD Comparisons

**Figure 1:**
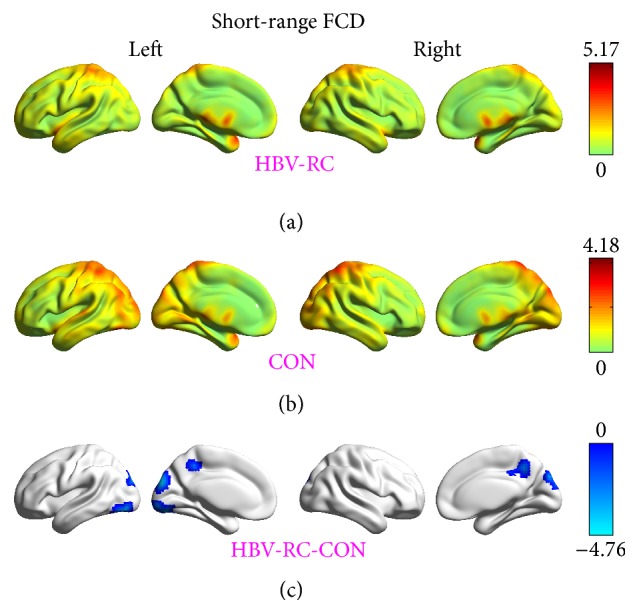
Short-range functional connectivity density (FCD) results. (a) Distribution of short-range FCD in the brains of patients with hepatitis B virus-related cirrhosis (HBV-RC). (b) Distribution of short-range FCD in the brains of the control (CON) group and significant differences (*t* scores) between groups. (c) The voxel-wise comparisons of short-range FCDs between two groups were conducted by using a one-way analysis of covariance (ANCOVA) with the gender, age, and average frame-wise displacement as nuisance covariates. The threshold was set at *p* < 0.05 with topological FDR corrected. The color bar represents the *t* score.

**Figure 2:**
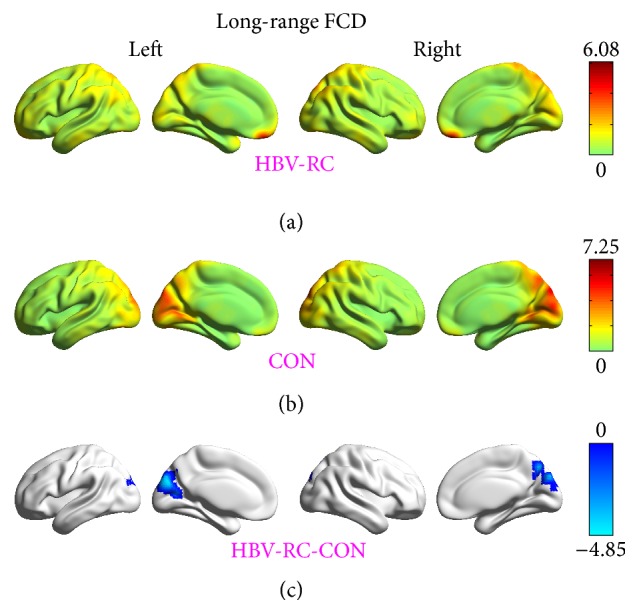
Long-range functional connectivity density (FCD) results. (a) Distribution of long-range FCD in the brains of patients with hepatitis B virus-related cirrhosis (HBV-RC). (b) Distribution of long-range FCD in the brains of the control (CON) group and significant differences (*t* scores) between groups. (c) The voxel-wise comparisons of long-range FCDs between two groups were conducted by using a one-way analysis of covariance (ANCOVA) with the gender, age, and average frame-wise displacement as nuisance covariates. The threshold was set at *p* < 0.05 with topological FDR corrected. The color bar represents the *t* score.

Compared with healthy controls, patients with HBV-RC showed significantly decreased short-range FCDs in the bilateral middle cingulum gyrus/precuneus, the bilateral cuneus, and the left lingual gyrus/inferior occipital gyrus (Figure 1, Table 2). Meanwhile, significantly decreased long-range FCDs in the bilateral cuneus/precuneus were also observed (Figure 2, Table 2).

### 3.3. Correlations between Long-Range FCD and PHES

**Figure 3:**
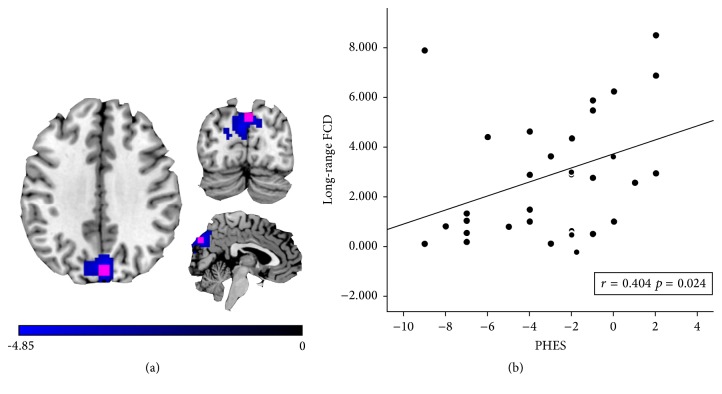
Results of Spearman correlation. (a) Significantly decreased long-range functional connectivity density (FCD) in the bilateral cuneus/precuneus. The region of interest (ROI) used in the correlation analysis was a 9 mm isotropic cube (marked in red) including 27 voxels which was centered at the Montreal Neurological Institute (MNI) coordinates of the local maxima. (b) Spearman correlations between altered long-range FCD in ROIs in the bilateral cuneus/precuneus and psychometric hepatic encephalopathy scores (PHES) of patients with HBV-RC.

In the HBV-RC group, Spearman correlations revealed that the ROIs with decreased long-range FCD in the bilateral cuneus/precuneus were significantly positively correlated with PHES (*r* = 0.404, *p* = 0.024; Figure 3). For ROIs with decreased short-range FCD, no regions correlated with PHES.

## 4. Discussion

The current resting-state FCD study indicated that, compared with the healthy controls, the patients with HBV-RC without overt HE had significantly reduced short-range FCD, reflecting the strength of the intraregional functional connectivity in the bilateral middle cingulum gyrus/precuneus, the bilateral cuneus, and the left lingual gyrus/inferior occipital gyrus. HBV-RC patients without overt HE also had reduced long-range FCD, reflecting the strength of the interregional functional connectivity, in the bilateral cuneus/precuneus. Overall, the regions with short- and/or long-range FCD reduction were primarily distributed in the bilateral precuneus, the middle cingulum gyrus, and the occipital visual-related cortices (the bilateral cuneus, the left lingual gyrus, and inferior occipital gyrus). Moreover, the decreased long-range FCD in the bilateral cuneus/precuneus was related to the performance of HBV-RC patients on the PHES test.

The precuneus is a central node in the human brain [28]. It is not only a critical hub of the default mode network [29] but also part of the primary visual network [30]. The precuneus is considered a high-level cognitive network and plays a significant role in visuospatial processing, episodic memory, reflections upon self, and awareness and conscious information processing [30]. Previous FCD studies [17, 19, 20] have demonstrated that the precuneus not only has the highest short-range FCDs but also shows significant increased long-range FCDs in healthy subjects. These findings [17, 19, 20] indicated that this region is an important hub that is both locally and remotely connected with other brain regions. Consistent with our observation that the precuneus exhibits decreased short- and long-range FCD in cirrhosis patients, previous studies have observed decreased functional connectivity related to this region using a seed-based functional connectivity method [31] and independent component analysis [14, 32]. Taken together, these findings indicate that the precuneus is closely related to the pathophysiology underlying cognitive deficits in cirrhotic patients.

A wide variety of evidence has demonstrated that the middle cingulate cortex is involved in many functions, including pain, negative affect, and cognitive control [33]. Recent neuroimaging study [34] suggests that the middle cingulate cortex plays a key role in the cognitive aspects of movement generation (i.e., intentional motor control). Motor symptoms, such as ataxia, tremor, and slowing of finger movements, are an obvious and major symptom in HE, even in patients without overt HE [35, 36]. The frequency of finger and hand tapping in cirrhosis patients with mild HE is also significantly correlated with the glucose metabolism of the motor area, including the cingulate gyrus [37]. Therefore, the observed decreased FCD in the middle cingulate cortex in patients with HBV-RC without overt HE suggests that the function of this region may be impaired.

The occipital cortices showed reduced FCD in the cuneus, the lingual gyrus, and the inferior occipital gyrus. These regions are key nodes of the ventral visual pathway and have been reported to play important roles in different visually associated functions [38]. Neuroimaging data [8, 9, 15] have consistently revealed that cirrhotic patients exhibit abnormal brain activity and functional connectivity within the visual association areas in the resting state. Our results also support previous studies indicating that cirrhotic patients display abnormalities in process of top-down modulation in visuospatial selective attention during event-related potential tests [39], decreased glucose metabolism in visual association brain regions in a previous positron emission tomography (PET) study [40], and impaired neural interaction between brain regions processing visual association information in a task fMRI study [41]. Thus, the decreased FCDs in the visual association areas observed in the current study may at least partially underlie the impairments in processing visual information that lead to some typical cognitive deficits in cirrhotic patients, such as deficits in visuospatial abilities, visual memory, and visuomotor.

An important finding of this study is the identification of positive correlations between cirrhotic patients' performance on PHES and their decreased long-range FCD in the bilateral cuneus/precuneus. The cuneus is essential for visual processing [41, 42]. The precuneus plays a pivotal role in the DMN [29]. The PHES, a neuropsychological tests battery that can reliably reflect most minimal HE-related neuropsychological impairments, primarily tests the domains of visual perception and construction, visual-spatial orientation, psychomotor speed, fine motor skills, concentration, attention, and working memory in cirrhotic patients [43]. Previous rs-fMRI studies [10, 11, 15] have also observed a significant correlation between abnormal resting-state brain activity or functional connectivity in cuneus/precuneus and neuropsychological tests. Thus, the impairment of the cuneus/precuneus in cirrhotic patients and its positive correlation with PHES are plausible. In addition, our findings reveal that decreased long-range FCD may serve as an alternative index for disease development.

Several limitations should be considered when explaining the findings. First, we used a 1.5T MR scanner. The image quality was rigidly controlled; however, to scan the entire brain, a relatively long TR of 3 s for multislice acquisitions was used. Under this relatively low sampling rate, physiological noise sources (e.g., respiratory and cardiac fluctuation effects) can be aliased into low-frequency BOLD MR signal fluctuations and may affect the final result. This should be taken with caution. In the future, relatively rapid acquisition (with a shorter TR) by using a 3.0T MR scanner [44], together with simultaneously recording the respiratory and cardiac cycle during the acquisition, may effectively reduce the these physiological noises. Second, in this preliminary study, our results are limited to a small number of participants, and a large cohort of participants and further group analysis are needed in future studies.

## 5. Conclusion

In conclusion, we investigated the map changes in brain functional connectivity in patients with HBV-RC without overt HE and determined that these patients had significant reductions in short- and/or long-range FCDs in brain areas distributed primarily in the bilateral precuneus, middle cingulum gyrus, and occipital visual-related cortices. Furthermore, the decreased long-range FCD in the bilateral cuneus/precuneus was related to the level of performance of HBV-RC patients on the PHES test. These findings may suggest aberrant functional connectivity density in HBV-RC patients prior to overt HE onset and may provide better insights into understanding the pathophysiological mechanisms underlying the cirrhotic-related cognitive impairment.

## Figures and Tables

**Table 1:** Demographics and clinical characteristics of patients with hepatitis B virus-related cirrhosis and healthy subjects.

	HBV-RC patients (*n* = 31)	Healthy controls (*n* = 30)	*Z* value	*p* values
Gender (male/female)	26/5	25/5	0.003	0.955
Age (years)	44.61 ± 10.01	43.73 ± 9.32	0.355	0.724
Education (years)	10.19 ± 3.57	11.80 ± 3.18	−1.854	0.069
Cigarettes	3.61 ± 6.19	3.60 ± 7.38	0.007	0.994
Head motion	0.127 ± 0.061	0.143 ± 0.074	−0.890	0.377
Child-Pugh A/B/C	17/10/4	—	—	—
Total serum bilirubin (IU/L)	38.30 ± 11.44	NP	—	—
Serum albumin (g/L)	35.37 ± 6.68	NP	—	—
Prothrombin time (s)	16.03 ± 1.97	NP	—	—
PHES (−15~5)^*∗*^	−2 (−9~2)	2 (−4~4)	−3.383	0.001^*∗∗*^

^*∗*^Means ± SD. ^*∗∗*^Significant difference. NP, not performed; PHES, psychometric hepatic encephalopathy score.

**Table 2:** FCD differences between patients with hepatitis B virus-related cirrhosis and healthy controls (patients < controls).

Brain regions	Cluster size (voxel)	BA	MNI coordinates			Peak *T* value
			*x*	*y*	*z*	
*Short-range FCD*						
Bilateral precuneus/middle cingulum gyrus	105	23, 31	3	−36	42	−4.759
Bilateral cuneus	267	19, 18	−3	−93	24	−4.556
Left lingual gyrus/inferior occipital gyrus	198	19, 18	−24	−81	−15	−4.467
*Long-range FCD*						
Bilateral cuneus/precuneus	431	19, 18, 7	3	−81	36	−4.851

FCD, functional connectivity density; BA, Brodmann area; MNI, Montreal Neurological Institute. The threshold was *p* < 0.05 with topological FDR corrected.
